# Safety, feasibility, and impact on the gut microbiome of kefir administration in critically ill adults

**DOI:** 10.1186/s12916-024-03299-x

**Published:** 2024-02-20

**Authors:** Vinod K. Gupta, Sanu Rajendraprasad, Mahmut Ozkan, Dhanya Ramachandran, Sumera Ahmad, Johan S. Bakken, Krzysztof Laudanski, Ognjen Gajic, Brent Bauer, Simon Zec, David W. Freeman, Sahil Khanna, Aditya Shah, Joseph H. Skalski, Jaeyun Sung, Lioudmila V. Karnatovskaia

**Affiliations:** 1 Microbiome Program, Center for Individualized Medicine, Mayo Clinic, Rochester, MN USA; 2 Division of Surgery Research, Department of Surgery, Mayo Clinic, Rochester, MN USA; 3 Division of Pulmonary and Critical Care Medicine, Mayo Clinic, Rochester, MN USA; 4 Department of Surgery, Mayo Clinic, Rochester, MN USA; 5 Section of Infectious Diseases, St Luke’s Hospital, Duluth, MN USA; 6 Department of Anesthesiology and Perioperative Care, Mayo Clinic, Rochester, MN USA; 7 Department of Internal Medicine, Mayo Clinic, Rochester, MN USA; 8 Department of Anesthesiology and Perioperative Care, Beth Israel Deaconess Medical Center, Boston, MA USA; 9 Department of Neurologic Surgery, Mayo Clinic, Jacksonville, FL USA; 10 Division of Gastroenterology, Department of Medicine, Mayo Clinic, Rochester, MN USA; 11 Division of Public Health, Infectious Diseases, and Occupational Medicine, Department of Medicine, Mayo Clinic, Rochester, MN USA; 12 Division of Rheumatology, Department of Medicine, Mayo Clinic, Rochester, MN USA

**Keywords:** Kefir, ICU, Critical illness, Probiotics, Gut microbiome, GMWI

## Abstract

**Background:**

Dysbiosis of the gut microbiome is frequent in the intensive care unit (ICU), potentially leading to a heightened risk of nosocomial infections. Enhancing the gut microbiome has been proposed as a strategic approach to mitigate potential adverse outcomes. While prior research on select probiotic supplements has not successfully shown to improve gut microbial diversity, fermented foods offer a promising alternative. In this open-label phase I safety and feasibility study, we examined the safety and feasibility of kefir as an initial step towards utilizing fermented foods to mitigate gut dysbiosis in critically ill patients.

**Methods:**

We administered kefir in escalating doses (60 mL, followed by 120 mL after 12 h, then 240 mL daily) to 54 critically ill patients with an intact gastrointestinal tract. To evaluate kefir’s safety, we monitored for gastrointestinal symptoms. Feasibility was determined by whether patients received a minimum of 75% of their assigned kefir doses. To assess changes in the gut microbiome composition following kefir administration, we collected two stool samples from 13 patients: one within 72 h of admission to the ICU and another at least 72 h after the first stool sample.

**Results:**

After administering kefir, none of the 54 critically ill patients exhibited signs of kefir-related bacteremia. No side effects like bloating, vomiting, or aspiration were noted, except for diarrhea in two patients concurrently on laxatives. Out of the 393 kefir doses prescribed for all participants, 359 (91%) were successfully administered. We were able to collect an initial stool sample from 29 (54%) patients and a follow-up sample from 13 (24%) patients. Analysis of the 26 paired samples revealed no increase in gut microbial α-diversity between the two timepoints. However, there was a significant improvement in the Gut Microbiome Wellness Index (GMWI) by the second timepoint (*P* = 0.034, one-sided Wilcoxon signed-rank test); this finding supports our hypothesis that kefir administration can improve gut health in critically ill patients. Additionally, the known microbial species in kefir were found to exhibit varying levels of engraftment in patients’ guts.

**Conclusions:**

Providing kefir to critically ill individuals is safe and feasible. Our findings warrant a larger evaluation of kefir’s safety, tolerability, and impact on gut microbiome dysbiosis in patients admitted to the ICU.

**Trial registration:**

NCT05416814; trial registered on June 13, 2022.

**Supplementary Information:**

The online version contains supplementary material available at 10.1186/s12916-024-03299-x.

## Background

Majority of critically ill patients experience gut dysbiosis, mainly diminished commensal gut microbiome diversity and expansion of pathogenic strains, often within hours of admission to the intensive care unit (ICU) [1–3]. Dysbiosis has been linked to increased susceptibility to hospital-acquired infections, organ failure, septic shock, and even mortality [4–12]. The precise mechanisms underlying the relationship between gut microbiome dysbiosis and these adverse outcomes remain poorly understood. However, prevailing theories suggest a potential cause involving an imbalanced gut microbiome that fosters colonization and proliferation of gut pathobionts, which has been considered as a risk factor for adverse outcomes in ICU patients [13]. Hence, these findings suggest that compromised gut microbiome diversity in critically ill patients is not only a marker of adverse outcomes but might also play a role in the progression and severity of their conditions.

Many standard therapeutic interventions employed in critical care unintentionally alter the commensal gut microbiome, often to the patient’s detriment. Common treatments such as antibiotics [14, 15], gastric acid suppression agents [16], opioids [17], antipsychotics [18], certain parenteral and enteral nutritional preparations [19], laxatives, and corticosteroids [20] not only deplete the commensal gut flora but also pave the way for pathogenic bacteria to thrive. Given the grave implications of gut dysbiosis in ICU settings, there is a mounting interest in devising interventions to counteract it. However, this endeavor has not been straightforward. For instance, recent clinical trials targeting a reduction in ventilator-associated pneumonia rates among critically ill patients through probiotic supplementation failed to yield positive results [21, 22].

Perhaps it is not surprising that probiotic supplementation did not significantly reduce ventilator-associated pneumonia rates in critically ill patients, as several studies have shown that probiotic supplementation does not necessarily change gut microbial diversity [23–28]. Traditionally, consuming a diet rich in fermented foods has been recognized as a way to promote good health, as suggested by the increased longevity of Bulgarian peasants, thought to be attributed to the benefits of lactic acid-producing bacteria in soured milk [29]. Several studies have corroborated that fermented foods, indeed, can enhance gut microbiome diversity [30–34]. One standout in this category is kefir, a well-known fermented milk beverage.

Kefir has been linked to a plethora of health benefits, ranging from anti-inflammatory and anti-oxidative effects to anti-cancer properties, and even antimicrobial activity against certain pathogenic bacteria [35–38]. These attributes naturally lead to the intriguing question: can kefir play a pivotal role in bolstering gut microbial diversity for critically ill patients? Yet, there is currently no substantial data regarding the effects of kefir on the microbiome diversity in such patients. Recognizing this gap, we embarked on a pilot study focused on the outcomes of oral kefir administration in critically ill patients admitted to the medical ICU setting. Our primary aim was to evaluate the feasibility, safety, and overall tolerance of kefir administration to patients. The secondary goal was to assess the impact of kefir on gut microbiome composition.

## Methods

### Study design

This study was conducted as an open-label phase I safety and feasibility trial, registered on ClinicalTrials.gov (NCT05416814). The kefir used was generously donated by Lifeway Foods® (Morton Grove, IL 60053). Approval for the study was granted by the Mayo Clinic Institutional Review Board (IRB# 20-005687), who deemed that no additional ethics approval was required for the study. IRB requested that the product should be treated as an investigational new drug by the Food and Drug Administration (FDA) to determine both the safety of administering kefir to critically ill patients and its feasibility, in terms of patient tolerance. Following a thorough review, the FDA approved the study. A secondary aim of our research was to explore shifts in the gut microbiome composition of ICU patients administered kefir during their ICU stay.

### Patient recruitment

We had pre-determined that a sample size of at least 50 patients would be sufficient to adequately assess the safety and feasibility of kefir administration in the ICU. Within the first 24 h of ICU admission, patients’ electronic health records (EHR) were reviewed for eligibility based on inclusion criteria. To qualify, patients had to be adults (> 18 years) with a functioning gastrointestinal (GI) tract, capable of tolerating either an oral diet or tube feeding administration, and anticipated to remain in the ICU for more than 48 h. On the other hand, the exclusion criteria encompassed the following: (i) a history of sustained immunosuppression (lasting at least 1 month) owing to medications like corticosteroids (≥ 10 mg daily), TNF-alpha inhibitors, monoclonal antibodies, or immunosuppressive anti-metabolites; (ii) compromised gut integrity, as indicated by conditions such as bowel resections, GI malignancy or bleeding, inflammatory bowel disease, intestinal obstructions, intra-abdominal hypertension, intestinal ischemia/reperfusion injuries, or secondary ileus; (iii) dairy intolerance or milk allergies; (iv) an extremely poor prognosis where survival through the treatment period was unlikely; and (v) pregnancy.

Patients or their proxy decision-makers were approached by one of the study team members. Those who agreed to participate provided verbal consent and signed the HIPAA Privacy Authorization form. All procedures performed in studies involving human participants were in accordance with the ethical standards set by the institutional and national research committees and with the 1964 Helsinki declaration and its later amendments or comparable ethical standards.

### Kefir product

Lifeway Foods® Kefir is a fermented milk beverage produced by adding kefir grains to milk or other liquids. These grains comprise a combination of yeast and bacteria (primarily *Lactobacillus* spp.), a protein-polysaccharide matrix (the most prominent component being kefiran, a polysaccharide composed of glucose and galactose). The beverage is enriched with eleven bacterial and one yeast cultures: *Bifidobacterium lactis*, *Lactobacillus lactis*, *Saccharomyces florentinus*, *Streptococcus diacetylactis*, *Lactobacillus acidophilus*, *Bifidobacterium bacterium longum* (or *Bifidobacterium longum*), *Lactobacillus casei*, *Lactobacillus reuteri*, *Lactobacillus plantarum*, *Lactobacillus rhamnosus*, *Bifidobacterium bacterium breve* (or *Bifidobacterium breve*), and *Leuconostoc cremoris*.

Each serving, equivalent to 240 mL or 8 oz., contains a concentration of 25 to 30 billion colony-forming units of these active and living cultures. This study utilized Lifeway Foods® Original Whole Milk Kefir Unsweetened and also Vanilla, Strawberry, and Mixed Berry flavors. Nutritional details of each serving are provided in Additional file 1: Table S1.

### Kefir administration to ICU patients

Lifeway Foods® Kefir was stored in a research pharmacy refrigerator at approximately 40 °F (4 °C). Each bottle, containing 960 mL (32 oz.) of kefir, had a unique identification number for tracking. Kefir doses were measured by a research pharmacist in three different aliquots (60 mL, 120 mL, or 240 mL) using a sterile syringe. We collaborated with the research pharmacy to build an EPIC EHR (Epic Systems Corporation, Madison, WI 53593) order set for kefir administration. We also included an order set for the bedside nurses instructing them to collect discarded stool samples into provided containers. Nurses were also directed to promptly notify the research team once a sample was ready for retrieval. Upon notification, a member of our study team would collect the sample and ensure its transfer to a dedicated freezer. All samples were frozen at −80 °C within 24 h of collection.

Following patient recruitment, kefir was either administered orally using a cup or delivered via a nasogastric tube. For the latter method, a tap water flush (up to 30 mL) was used afterwards. Study participants received kefir following an ascending dosing schedule. Initially, a 60 mL dose was provided. If patients showed good tolerance, the dosage was increased to 120 mL within the subsequent 12 h. Barring any adverse reactions (e.g., nausea, vomiting, bloating, or abdominal discomfort), a 240-mL dose was given 12 h later with all remaining doses being 240 mL (a standard serving dose) every 24 h. However, if any intolerance was observed, the dosage was reverted to the last well-tolerated amount.

### Outcome measures

The primary objective of this study was to assess the safety and feasibility of administering kefir to ICU patients. Safety was defined based on the incidence of adverse events directly attributable to kefir administration. We specifically observed for bloating, vomiting, aspiration, diarrhea, interactions with medications or tube feedings, bacteremia, or fungemia resulting from any of the bacterial or yeast species present in kefir. To evaluate the safety of kefir administration, daily communication with the bedside nurse and a thorough review of the electronic health record (EHR) were undertaken. Feasibility was determined as delivery of more than 75% of the prescribed kefir doses, provided patients were permitted oral or feeding tube administration. The patients were followed until they were discharged from the hospital.

### Stool sample collection, DNA extraction, and shotgun metagenome sequencing

For gut microbiome analysis, we aimed to collect the first stool sample as early as possible following a patient’s admission to the ICU, ideally within 72 h of the first kefir dose. This timing was contingent on whether the patient had a bowel movement within this period. Considering the initial dosing regimen of kefir, its impact on the gut microbiome at this early stage was anticipated to be minimal. The second stool sample was planned for collection after at least 72 h of administering the full dose of kefir to the patients.

Following study completion, all stool samples were sent to the University of Minnesota Genomics Center for DNA extraction and sequencing. Fecal DNA was extracted using Qiagen’s DNeasy 96 PowerSoil Pro QIAcube HT Kit following the manufacturer’s instructions (QIAGEN, Germantown, MD, USA) and was quantified using a NanoDrop-8000 UV-Vis Spectrophotometer (ThermoScientific, Wilmington, DE, USA) and PicoGreen assays. Samples were then loaded onto the QIAcube HT, an automated DNA extraction instrument. DNA was quantified using Qubit before preparing sequencing libraries using the Nextera XT protocol. Metagenomic sequencing libraries were loaded onto an Illumina Novaseq 6000 sequencer or smaller-scale Illumina sequencing instrument using the same 2 × 150 bp chemistries, targeting 8 M paired-end reads per sample.

### Quality filtration of sequenced reads

Metagenome reads were processed using an in-house quality-filtration pipeline, which uses Trimmomatic v0.39 [39] and Bowtie2 v2.3.3.1 [40] for the removal of low-quality read bases and human reads, respectively. Trimmomatic v0.39 was run with parameters LEADING:3, TRAILING:3, and MINLEN:60. Illumina adapter sequences were removed, and trimmed non-human reads shorter than 60 bp in nucleotide length were discarded. Potential human contamination was filtered by removing reads that aligned to the human genome (reference genome hg38).

### Taxonomic profiling of stool metagenomes

Taxonomic profiling was performed using the MetaPhlAn3 v3.0.13 phylogenetic clade identification pipeline with default parameters [41]. Briefly, MetaPhlAn3 classifies metagenome reads to taxonomies based on a database (mpa_v30_CHOCOPhlAn_201901) of clade-specific marker genes derived from ~ 100,000 microbial reference genomes (corresponding to ~ 99,500 bacterial and archaeal and ~ 500 eukaryotic genomes). Microbes of viral origin and those that were labeled as either unclassified or unknown were excluded from further analyses. Afterward, microbiome profiles were normalized using total sum-scaling (TSS) normalization to get the relative abundances (i.e., proportions) of microbial taxonomic ranks.

### Measuring gut microbiome diversity

The overall ecology of gut microbiomes was evaluated by calculating α-diversity (Shannon Index and species richness) and *β*-diversity (Bray–Curtis distance between all sample pairs). The R package “vegan” v2.6.4 was used to calculate the Shannon Index and species richness based on the untransformed species’ relative abundance profiles of each stool metagenome. α-diversities between two timepoints were compared using the Wilcoxon signed-rank test. The R packages “ade4” v1.7-22 and “vegan” v2.6.4 were used to perform principal coordinate ordination analysis (PCoA) with Bray–Curtis dissimilarity as the distance measure on the arcsine square root-transformed relative abundances of the microbial species identified by MetaPhlAn3.

### Permutational multivariate analysis of variance based on taxonomic composition of microbial communities

Bray–Curtis distance matrices based on arcsine, square-root transformed relative abundances of microbial taxa species in stool metagenomes were generated using the R “vegan” package v2.6.4. A permutational multivariate analysis of variance (PERMANOVA) was performed on the distance matrix using the “adonis2” function. *P*-values for the test statistic (pseudo-F) were based on 999 permutations to assess the contribution of timepoint to the total variance in gut microbial community composition, while random permutations were constrained within subjects by using the “strata” option. Both marginal (i.e., univariate analysis) and adjusted (i.e., multivariate analysis controlling for multiple covariates simultaneously) models were used to evaluate percent variance and significance of associations between gut microbiome composition and timepoint.

### GMWI calculation

The Gut Microbiome Wellness Index (GMWI) [previously called the Gut Microbiome Health Index (GMHI)] is a stool metagenome-based indicator for monitoring health [42]. GMWI for this study was derived using a modified version of the original GMWI script on species-level gut microbiome taxonomic profiles. This index is based on 42 gut microbial species prevalent in healthy individuals (those without disease) or in non-healthy individuals (those clinically diagnosed with a disease). These microbial species (Additional file 1: Table S2) were identified from the gut microbiomes (analyzed through stool shotgun metagenomes) of 5547 healthy and 2522 non-healthy subjects. A positive (negative) value of GMWI for a given stool metagenome sample suggests that microbes associated with a healthy, disease-free state are more (less) abundant than microbes associated with a non-healthy, disease-harboring state, and a zero value indicates that there is an equal balance of both species sets. Therefore, the GMWI can be interpreted as the extent to which a microbiome sample contains a higher collective abundance of health-prevalent species relative to health-scarce species.

We posited that kefir administration could enhance the gut health of critically ill patients, gauged through GMWI. To assess the impact of kefir on the gut microbiome in ICU patients, we examined the GMWI progression from timepoint T1 to T2. Our hypothesis anticipated a specific directional shift, namely an increase in GMWI. Accordingly, we used the one-sided Wilcoxon signed-rank test, a choice aligned with our directional hypothesis, to analyze the data. This method was selected as it targets changes in a defined direction, as opposed to measuring a general difference.

### Quantification of fold change in GMWI species from timepoint 1 to timepoint 2

We focused on the aforementioned (health-prevalent and health-scarce) microbial species involved in the GMWI calculation (as listed in Additional file 1: Table S2). For each species, we computed the fold change as the ratio of the geometric mean of relative abundances at timepoint 2 to that at timepoint 1. Since a geometric mean cannot be computed if a species is absent (i.e., relative abundance = 0) in all samples at either timepoint, we replaced all zero values with a small pseudo-count of 3.6 × 10^−8^. We then conducted a permutation test to identify any statistically significant differences in fold changes between the two timepoints. In our approach, the class labels (here, the timepoint designations) underwent random permutation to establish the null distribution and fold changes were computed. Over 10,000 iterations, we counted occurrences where the fold changes were equal to or more extreme than the observed value to derive a *P*-value. We deemed fold changes with *P* < 0.05 to be statistically significant.

## Results

### Patient selection, safety monitoring, and stool sample collection

**Fig. 1:**
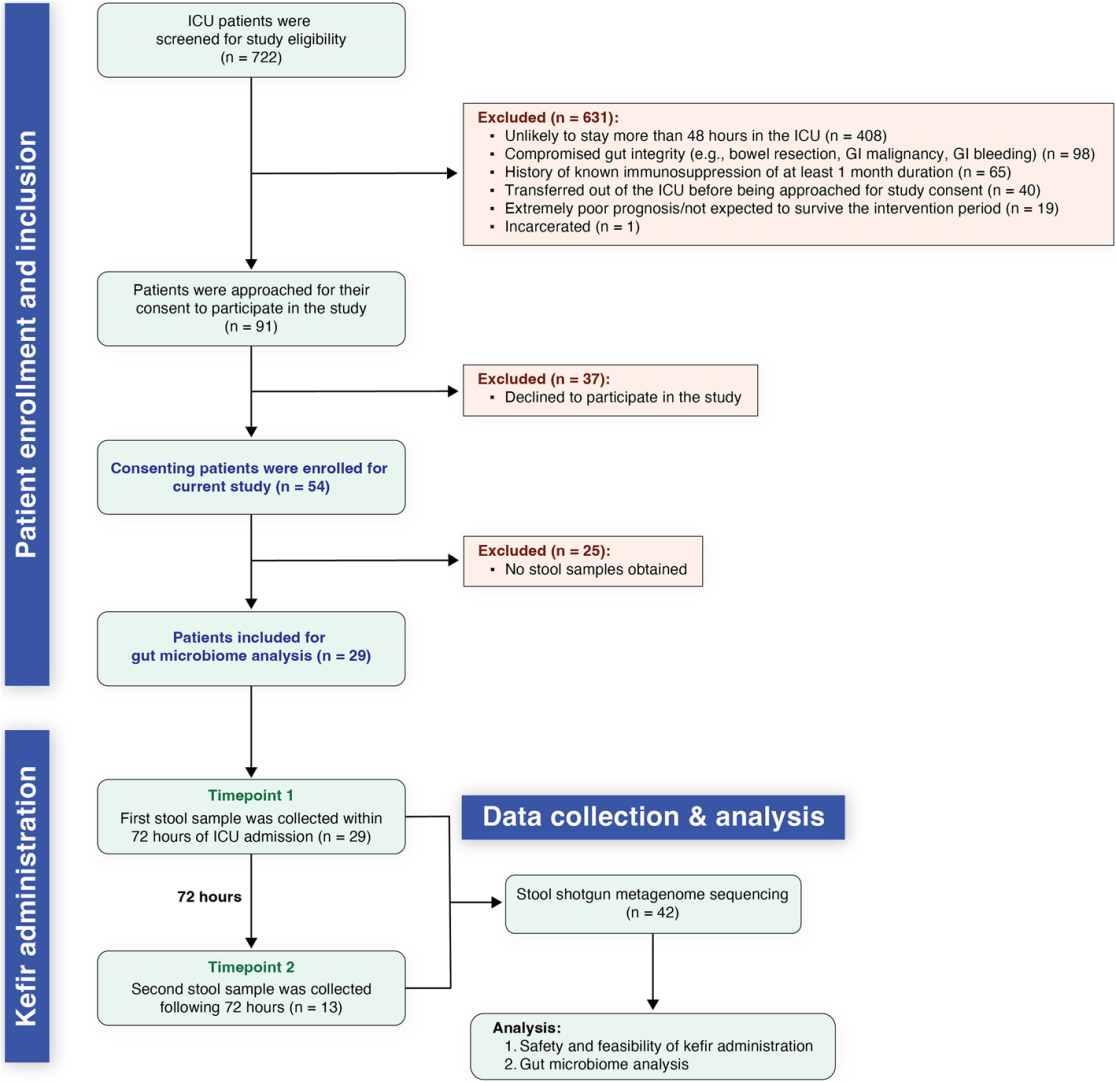
Patient selection and stool sample collection for gut microbiome analysis. Of an initial 722 ICU admissions screened, 54 patients were enrolled in the current study based on our criteria. For gut microbiome analysis, stool samples were collected from patients after kefir administration; however, 25 of the 54 participants did not have bowel movements during their ICU stay and were unable to provide a stool sample. For the remaining 29 patients, an initial stool sample was collected within 72 h of ICU admission (from all 29 patients), with a subsequent sample gathered after kefir administration (from 13 patients), contingent on bowel activity

We screened the health records of 722 patients admitted to the ICU for various reasons from July 2022 to February 2023 for potential inclusion in our study (Fig. 1). Of this cohort, 54 patients met our criteria and were enrolled, while 668 were excluded based on specified criteria detailed in Fig. 1. The enrolled patients had a mean age of 64.6 years (s.d. = 15.3) and a mean BMI of 34.9 (s.d. = 13.0) at the point of ICU admission (Table 1). The demographic breakdown revealed that 39% (21 out of 54) were female, and a substantial majority (98%, or 53 out of 54) identified as white. Furthermore, 87% (47 out of 54) of the participants were on at least one antibiotic regimen during their ICU stay, and 78% (42 of 54) received antibiotics with anaerobic coverage (Table 1). The primary comorbidities and surgical histories as well as clinical reasons for all patients' admission to the ICU are detailed in Table 1. The most frequent diagnoses included respiratory failure (non-COVID related) and shock. Upon admission to the ICU, the average Apache score (used as an indicator of disease severity and mortality estimate, with a range of 0–71; a higher score indicates a higher mortality risk) was 39.8 (s.d. = 22.0). This metric was incorporated primarily to describe the clinical population of our study, in line with its focus on safety and feasibility. The median duration of stay in the medical intensive care unit (MICU_LOS) was 6 days, while the overall hospital stay averaged at 11 days (Table 1). Additional demographic and ICU stay details for the 13 patients with 2 stool samples are also provided in Table 1.

**Table 1:** Demographic and clinical profile of study participants

Variable	All patients (*n* = 54)	Patients with 2 stool samples (*n* = 13)
Age (mean ± s.d., years)	64.6 ± 15.3	66.6 ± 15.7
Biological sex		
Male (*n*, %)	33 (61.1)	7 (54%)
Female (*n*, %)	21 (38.9)	6 (46%)
BMI (mean ± s.d., kg/m^2^)	34.9 ± 13.0	36.8 ± 17.2
Race		
White (*n*, %)	53 (98.1)	12 (92%)
American Indian/Alaskan Native (*n*, %)	1 (1.9)	1 (8%)
Comorbidities		
Coronary artery disease (*n*)	11	3
Peripheral vascular/arterial disease (*n*)	5	1
Atrial fibrillation (*n*)	14	4
Heart failure (*n*)	24	5
Deep venous thrombosis (*n*)	5	2
Diabetes (*n)*	26	9
Hypothyroidism (*n*)	6	3
Chronic kidney disease (*n*)	14	3
Hypertension (*n*)	22	7
Reflux (*n*)	5	2
Asthma (*n*)	2	
Chronic obstructive pulmonary disease (*n*)	14	1
Interstitial lung disease (*n*)	5	1
Obstructive sleep apnea (*n*)	12	3
Cirrhosis (*n*)	6	1
Crohn's disease (*n*)	1	
Chronic pancreatitis (*n*)	1	
History of any cancer (*n*)	9	
Arthritis (*n*)	1	
Parkinson’s disease (*n*)	1	1
Epilepsy (*n*)	2	2
Cognitive impairment (*n*)	3	1
History of stroke (*n*)	4	2
Pressure ulcer/osteomyelitis (*n*)	4	1
Neurogenic bladder (*n*)	2	
Active smoking (*n*)	12	3
Alcohol abuse (*n*)	7	1
Active drug use (*n*)	5	
Surgical history		
Splenectomy (*n*)	1	
Polypectomy (*n*)	2	1
Variceal banding/clipping (*n*)	3	
Bariatric surgery (*n*)	1	
Cholecystectomy (*n*)	1	
Cystoscopy with stenting (*n*)	3	
Arthroplasty (*n*)	3	1
Subtotal colectomy/colostomy/ileostomy (*n*)	4	
Lumbar spine fusion (*n*)	2	1
Toe or below the knee amputation (*n*)	4	2
Ureteral stenting (*n*)	1	
Implantable cardioverter-defibrillator/pacemaker (*n*)	6	1
Coronary artery bypass/cardiac catherization (*n*)	8	2
Carotid endarterectomy (*n*)	2	1
Cataract surgery (*n*)	5	
Apache^a^ score (mean ± s.d.)	39.8 ± 22.0	52.1 ± 24.9
MICU_LOS^b^ (median days, Q1, Q3)	6 (3, 10)	17.5 (10.5, 22.5)
Hospital_LOS^c^ (median days, Q1, Q3)	11 (6, 23)	23 (20, 46)
Reason for ICU admission		
Pneumonia, non-COVID (*n*)	17	6
COVID pneumonia (*n*)	4	4
Hypercapnic respiratory failure (*n*)	6	1
Hypoxemic respiratory failure, non-infectious (*n*)	12	2
Shock (septic, hemorrhagic) (*n*)	22	4
Urinary tract infection, complicated (*n*)	3	
Diabetes complications (*n*)	4	2
Atrial fibrillation with rapid response (*n*)	2	
Altered mental status/encephalopathy (*n*)	4	
Electrolyte derangements (*n*)	5	1
Substance overdose/withdrawal (*n*)	4	
Acute kidney injury (*n*)	14	
Cardiac arrest/non-ST-elevation myocardial infarction (*n*)	4	1
Heart failure exacerbation (*n*)	6	1
Hypertensive emergency (*n*)	2	
Pancreatitis (*n*)	2	1
Cellulitis (*n*)	3	2
ICU treatments		
Inotropes (*n*)	38	12
Mechanical ventilation (*n*)	33	13
Sedation (*n*)	31	12
Tube feedings (*n*)	26	12
Paralytics (*n*)	2	0
Antibiotics use		
Any antibiotic given^d^ (*n*, %)	47 (87)	13 (100%)
Anaerobic antibiotic given^e^ (*n*, %)	42 (78)	12 (92%)
None (*n*, %)	7 (87)	0

^a^Apache, Acute Physiology and Chronic Health Evaluation

^b^MICU_LOS, Length of stay in the Medical Intensive Care Unit

^c^Hospital_LOS, Length of stay in the hospital

^d^Antibiotics included ceftriaxone (*n* = 26), vancomycin (*n* = 26), piperacillin-tazobactam (*n* = 23), cefepime (*n* = 15), azithromycin (*n* = 14), metronidazole (*n* = 14), doxycycline (*n* = 5), cefazolin (*n* = 3), oxacillin (*n* = 2), trimethoprim-sulfamethoxazole (*n* = 2), rifaximin (*n* = 2), levofloxacin (*n* = 1), and clindamycin (*n* = 1)

^e^Piperacillin-tazobactam, metronidazole, trimethoprim-sulfamethoxazole, levofloxacin, and clindamycin

### Kefir administration is both feasible and safe for critically ill patients

We administered kefir through a nasogastric tube for 52% (28 out of 54) of the patients, while the rest ingested it orally. We did not encounter any instances that necessitated reverting to a previous dose level; therefore, no dose adjustments were required. Eighty percent (43 of 54) of patients received at least three kefir doses; the main reason for discontinuation was transfer to the ward or transitioning to comfort care. From the 393 doses we ordered, 91% (359 of 393) were administered, which aligned with our feasibility goal as outlined in the “Methods” section. Overall, there was a median of 56 h (interquartile range [IQR]: [28, 83]) from the first kefir dose to the collection of the first stool sample and a median of 160 h (IQR: [144, 193]) from the first dose to the second sample collection. There were instances where kefir administration was interrupted (due to procedures or changes to NPO status) and then resumed. Despite several instances where the time to the first stool sample exceeded our target window of 72 h, we chose to analyze all available data, considering the pilot nature of our study. Patients consumed kefir for a median duration of 3 days (IQR: [2, 7]), with 4 patients being on kefir for 2 weeks and 2 patients receiving it for 4 weeks.

Of the 54 study participants, four dropped out. One patient reported bloating, which was later found to be related to a prior diagnosis of chronic mesenteric ischemia. Another patient expressed a dislike for the taste of the unflavored kefir. Consequently, we introduced flavored varieties; despite this adjustment, two more individuals still found the taste unappealing.

Two individuals experienced diarrhea, but its link to kefir was unclear given concurrent laxative use. Many critically ill patients have difficulty with bowel movements due to many factors, including opioid use. As a common practice, a bowel regimen (e.g., laxatives) is often introduced if a patient has not had a bowel movement for several days. In our study, both patients who developed diarrhea were on a bowel regimen while also receiving kefir. Diarrhea is a frequent occurrence in the ICU, typically triggered by tube feeds, among other factors. Notably, both patients in our study who experienced diarrhea were receiving tube feeds. Given these circumstances, it was challenging to definitively attribute the cause of diarrhea. The absence of a control arm in this pilot study further complicates the isolation of the cause. Importantly, no cases of bacteremia, fungemia, or deaths were associated with kefir. Thus, our findings indicate that administrating kefir to critically ill patients is both feasible and safe.

### Critically ill patients show significant changes in gut microbiome composition in the ICU

**Fig. 2:**
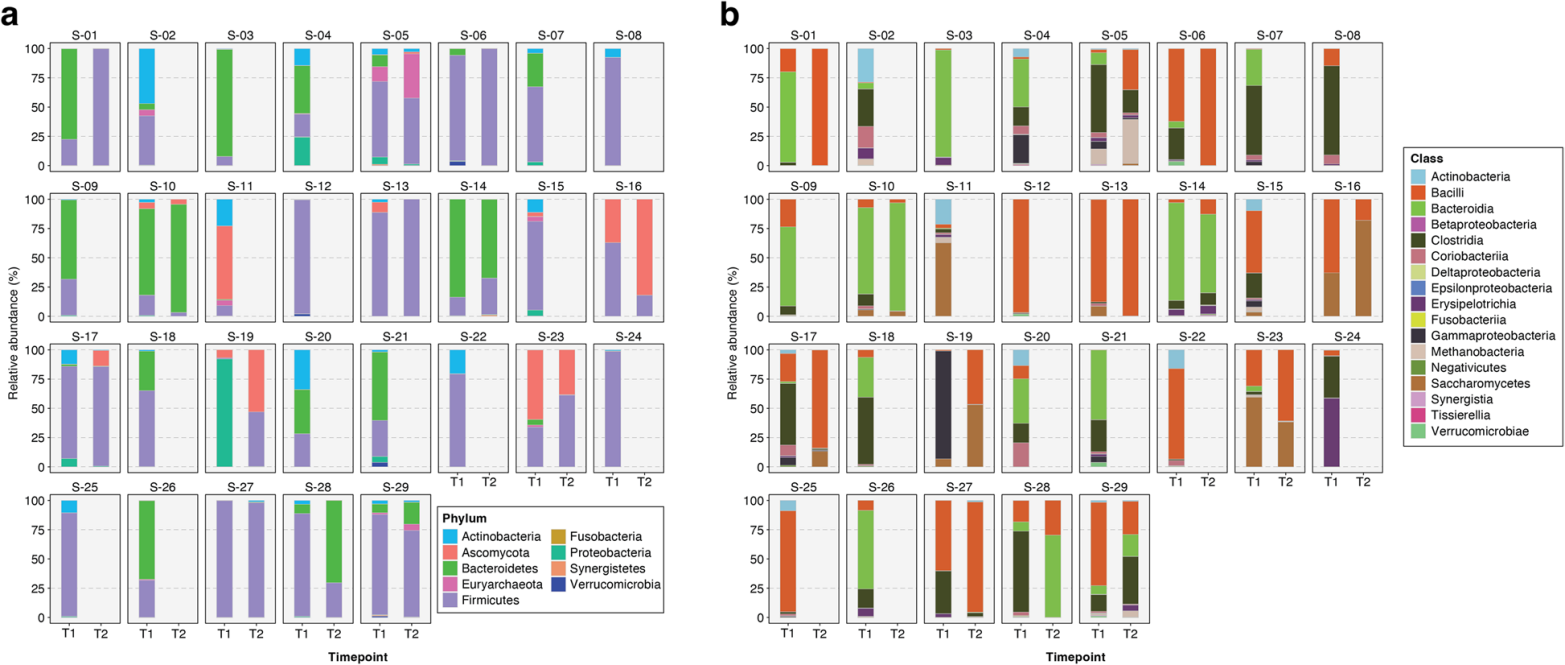
Stacked bar plots showing the relative taxonomic abundances in the gut microbiomes of 29 ICU patients at two timepoints: T1 (within 72 h of ICU admission) and T2 (after 72 h). In general, the most abundant **a** phyla are Firmicutes and Bacteroidetes, while Bacilli and Bacteroidia dominate the **b** class level

In the gut microbiomes (stool shotgun metagenomes) of critically ill patients, we found that Bacteroidetes and Firmicutes were the most abundant phyla at both timepoint 1 (T1, *n* = 29) and timepoint 2 (T2, *n* = 13) (Fig. 2a), while Bacteroidia and Bacilli were the most abundant classes (Fig. 2b). When patients were admitted to the ICU, the average number of phyla and classes in their gut microbiome was 4.9 (s.d. = 1.5) and 8.5 (s.d. = 2.6), respectively. However, this number decreased significantly during their stay in the ICU, likely due to the routine administration of antibiotics to patients in a medical ICU setting. By T2, the average count had declined to 4.0 phyla (s.d. = 1.8) and 5.9 classes (s.d. = 3.5).

**Fig. 3:**
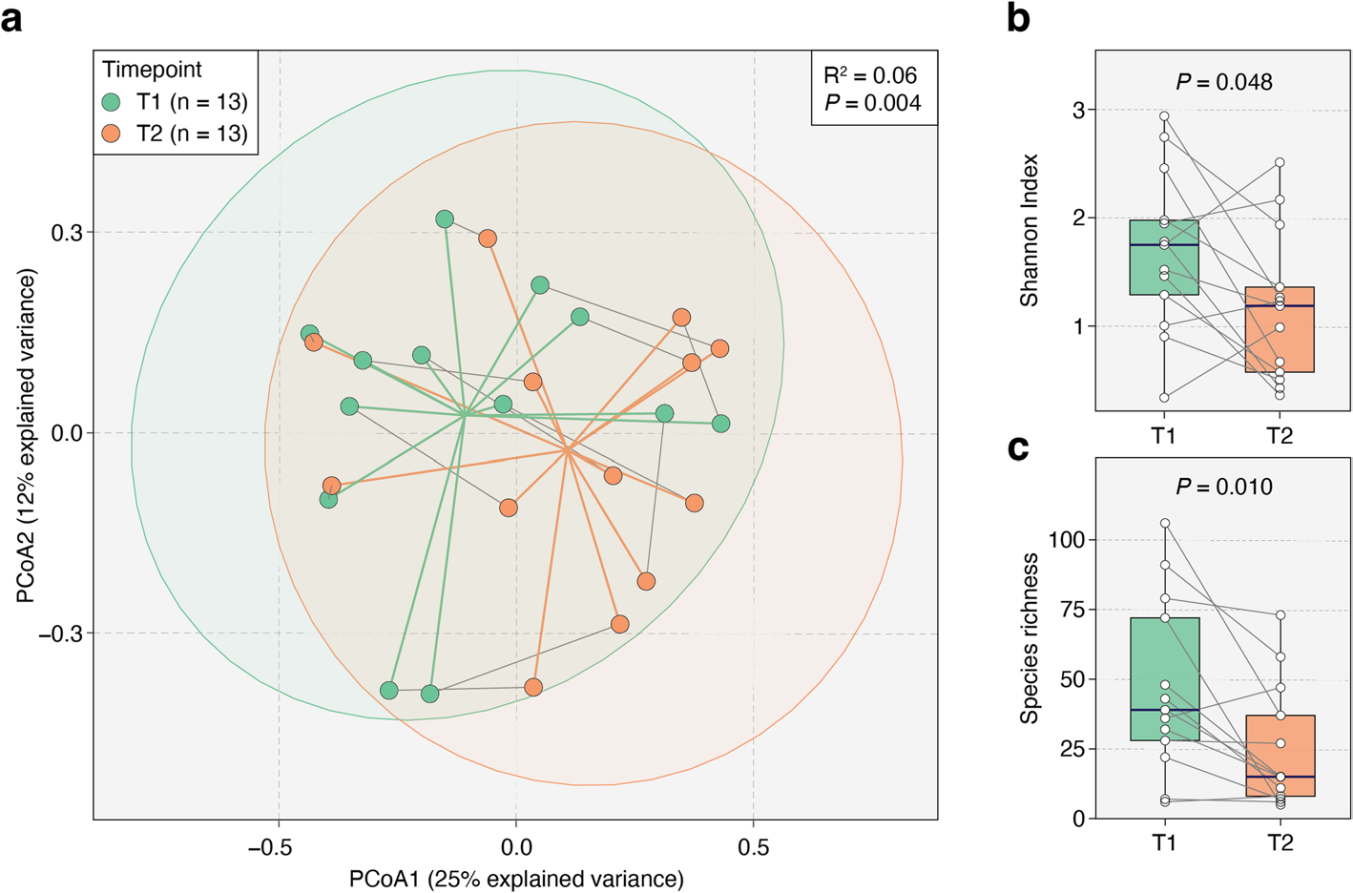
Gut microbiome diversity alterations in 13 ICU patients across two timepoints. **a** Principal coordinate analysis (PCoA) ordination plots of gut microbiome samples from patients at two timepoints (26 total samples). A significant shift in gut microbiome composition between the timepoints was identified by our PERMANOVA analysis (*R*^2^ = 0.06, *P* = 0.004). Points represent samples from T1 (green) and T2 (orange). Gray solid lines connect samples from the same patient, with green and orange lines marking the centroids of T1 and T2 samples, respectively. Ellipses correspond to 95% confidence regions. **b**, **c** T2 samples showed significant reductions in both species-level Shannon Index (*P* = 0.048) and richness (*P* = 0.010) compared to T1. *P*-values were obtained using the two-sided Wilcoxon signed-rank test

On gut microbiomes of stool samples collected from the same patient at two distinct timepoints (*n* = 13), we conducted a Permutational multivariate analysis of variance (PERMANOVA) analysis on Bray–Curtis distances to evaluate the impact of timepoint on the variance in gut microbial communities of critically ill patients (“Methods”). After adjusting for intra-subject variation, we found that the timepoint accounted for 6% of the total variance in gut microbial communities (*P* = 0.004, PERMANOVA; Fig. 3a). This result suggests that the ICU environment has a significant impact on the overall gut microbiome composition longitudinally.

In paired gut microbiome samples (collected within 72 h) from the 13 patients, we observed significant reductions in both the Shannon Index (*P* = 0.048, two-sided Wilcoxon signed-rank test; Fig. 3b) and species richness (*P* = 0.010, two-sided Wilcoxon signed-rank test; Fig. 3c) from T1 to T2. As mentioned above, these changes are most likely attributable to the antibiotics given during the ICU stay.

### Microbial species found in kefir display varying engraftment in patients’ guts

**Fig. 4:**
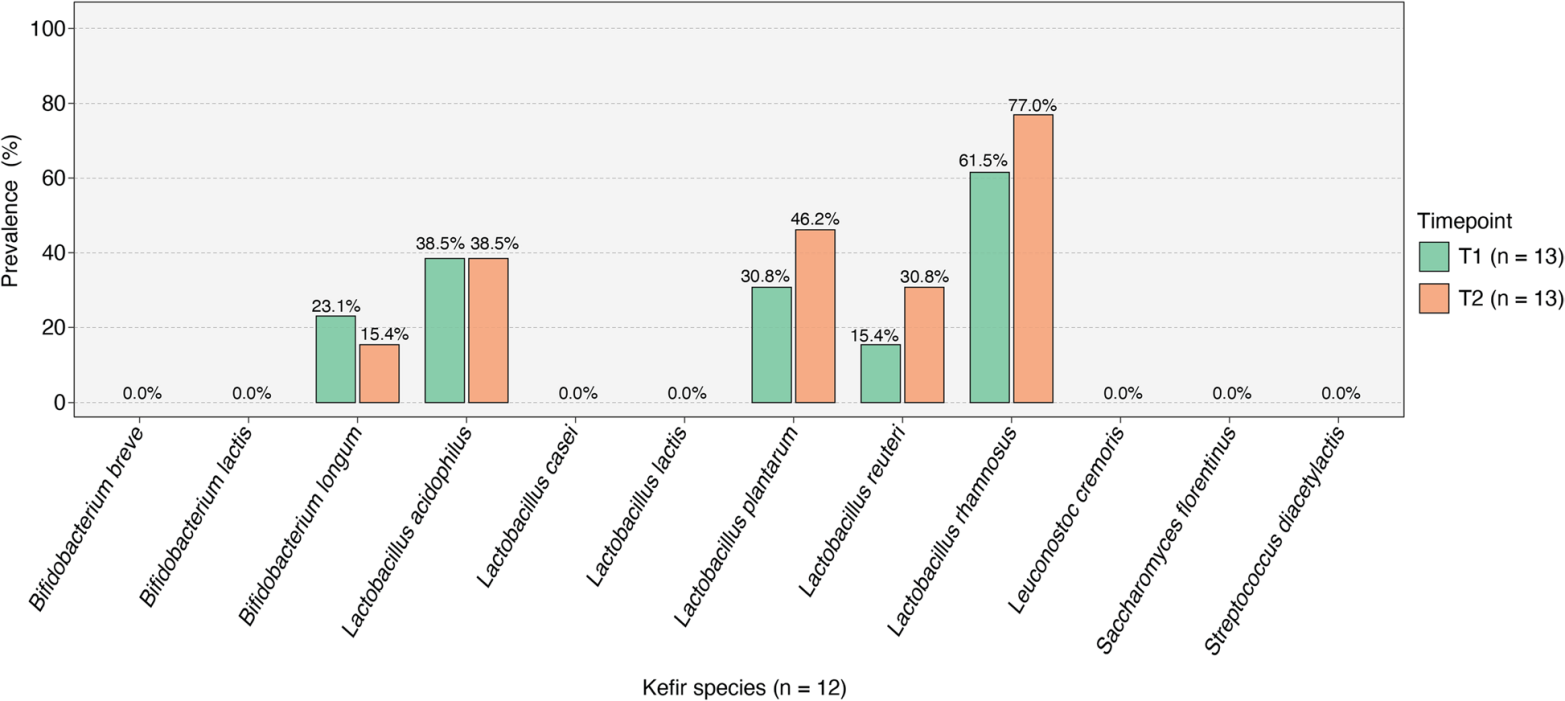
Microbial species from kefir exhibit different patterns of gut engraftment. T1 and T2 prevalences of the 12 microbial species found in kefir. There was an increased prevalence of three *Lactobacillus* species in ICU patients (*Lactobacillus plantarum*, *Lactobacillus reuteri*, and *Lactobacillus rhamnosus*). However, the prevalence of *Lactobacillus acidophilus* remained unchanged, and *Bifidobacterium longum* was detected less frequently at T2. Seven species were not detected in any of the analyzed stool samples

After analyzing α-diversity in the gut microbiomes of the 13 patients at two distinct timepoints, we found that kefir intake at a dose of one serving a day did not increase overall microbial diversity. However, when we delved deeper to explore changes in the prevalence of 12 specific microbial species known to be in kefir (Fig. 4), a few intriguing patterns emerged. Over a brief 72 h period between T1 and T2, we observed a significant increase in the prevalence (i.e., the proportion of samples where a specific microbe was detected) of three *Lactobacillus* species: *Lactobacillus plantarum*, *Lactobacillus reuteri*, and *Lactobacillus rhamnosus*. Conversely, the prevalence of *Lactobacillus acidophilus* remained the same, and *Bifidobacterium longum* was less frequently detected at T2 than T1. Notably, of the 12 kefir species, seven were undetected in all patients at both timepoints, indicating their complete lack of engraftment in the gut.

### Critically ill patients exhibit improvement in their gut microbiome following kefir supplementation in the ICU

**Fig. 5:**
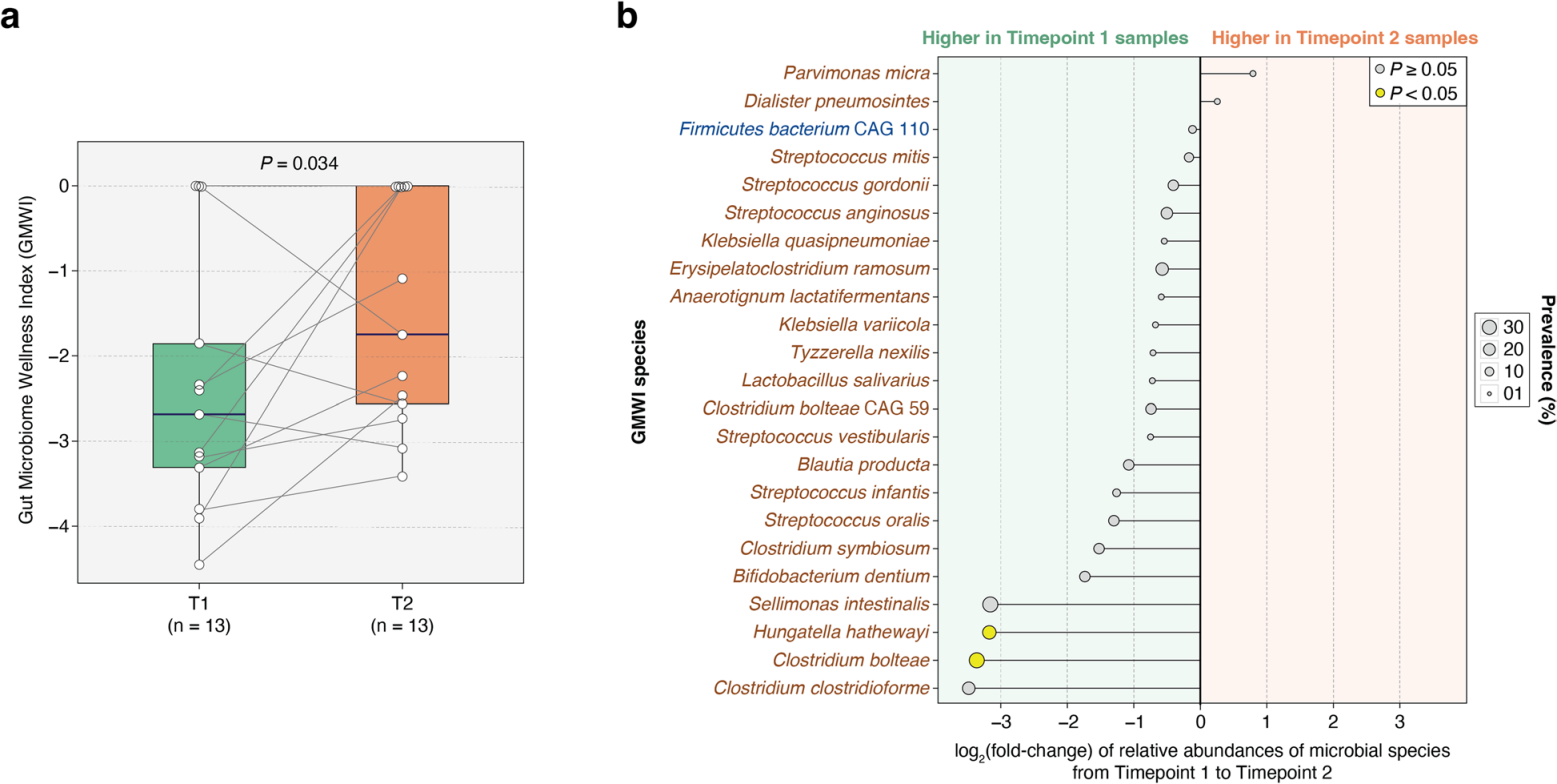
Changes in gut microbiome wellness and GMWI species abundances in ICU patients. **a** GMWI increased significantly (*P* = 0.034, one-sided Wilcoxon signed-rank test) from T1 to T2. **b** The chart represents the fold change in relative abundances of GMWI species between T1 and T2, with the names of health-prevalent species depicted in blue (*n* = 1) and those of health-scarce species (*n* = 22) in brown. Notably, two health-scarce species, *Hungatella hathewayi* and *Clostridium bolteae*, exhibited a statistically significant decrease in relative abundance by T2 (*P* < 0.05, permutation test). The black horizontal lines indicate the extent of fold change, while the size of each circle reflects the prevalence of the respective GMWI species among the 26 stool samples gathered across both timepoints

In our study, we hypothesized that kefir administration would improve gut health in critically ill patients. The decision to administer kefir in the medical ICU was fundamentally based on the hypothesis that it would yield beneficial effects. Therefore, to gain deeper insights into the benefits of kefir supplementation on the gut microbiome of ICU patients, we analyzed the Gut Microbiome Wellness Index (GMWI) transition from T1 to T2. As previously mentioned in our earlier work [35], GMWI is a superior indicator of overall gut health than ecological indices of diversity. Interestingly, there was a significant rise in GMWI at T2 (*P* = 0.034, one-sided Wilcoxon signed-rank test; and Fig. 5a), which indicates an improvement in the gut microbiome of patients during their stay in the ICU.

As GMWI factors in both health- and disease-associated microbial species in its calculation, we investigated the change in the relative abundance of each GMWI species (Additional file 1: Table S2) between T1 and T2. Of the total 42 GMWI species (15 health-prevalent and 27 health-scarce), only 23 were observed in the gut microbiomes of the 13 patients whose samples were available at both timepoints (Fig. 5b). From this subset, only one was a health-prevalent species (*Firmicutes bacterium* CAG110), with the remaining 22 being health-scarce. Intriguingly, all GMWI species exhibited a fold change (in relative abundance) decline from T1 to T2 except for two species i.e., *Parvimonas micra* and *Dialister pneumosintes*, which showed an increase in fold change (Fig. 5b). However, for most species, their changes in relative abundance from T1 to T2 were not statistically significant (*P* ≥ 0.05, permutation test). Only two specific species, *Hungatella hathewayi* and *Clostridium bolteae*, did show a statistically significant reduction at T2 (*P* < 0.05, permutation test; and Fig. 5b).

## Discussion

In this phase 1 study, we demonstrated that administering kefir is both safe and feasible for critically ill patients who have a functional gastrointestinal system and were not on sustained immunosuppression prior to admission. Kefir administration was well tolerated with minimal or no side effects. Though our analyses showed that microbial diversity and species richness in their gut microbiomes did not significantly increase following kefir intake in the ICU (Fig. 3b–c), we observed an increase in the Gut Microbiome Wellness Index (GMWI) from timepoint 1 (T1) to timepoint (T2) (Fig. 5a). For reference, GMWI serves as an indicator of the overall health of the gut microbiome [42].

At the time we designed and performed our investigation, there were no published reports on the safety and feasibility of administering kefir to critically ill patients in the intensive care setting. Lillie et al. recently presented a conference abstract that discussed the results of a retrospective study where they provided 120 mL of kefir twice daily to severely injured patients, including patients with burn injuries who were receiving enteral nutrition [43]. However, the study primarily aimed to compare rates of *C. difficile* infection, catheter-associated urinary tract infection, and central line-associated bloodstream infection between patients who received kefir and those who did not. Their results showed no significant difference in the rates of these hospital-acquired infections between the two groups. Of note, there is an ongoing randomized control trial investigating whether administration of 120 mL kefir three times daily may impact incidence of diarrhea and *C. difficile* infection in patients hospitalized on the general medicine ward (NCT02707198).

In examining the gut microbiome profiles from the 13 patients across two timepoints, we found that upon ICU admission, all critically ill patients displayed declining microbial diversity and species richness during their ICU stay (Fig. 3b, c). Loss of gut microbiome diversity in the critically ill is linked with depletion of short-chain fatty acid biosynthesis, conferring an increased risk of developing hospital-acquired infections [4, 5, 44]. Patients who received anaerobic antibiotic coverage in the ICU had increased concentration of Enterococci in their stool, which translated into higher numbers of organ failures and mortality rates [13, 45]. An investigation that examined fecal microbiome before and after cardiac surgery reported a significant increase in potentially pathogenic bacterial species following admission, and lower bacterial diversity was associated with longer hospitalization [46]. This observation has also been noted among the neurocritically ill, with an abundance of Enterobacteriaceae associated with a higher modified Rankin Scale at discharge, and a 92% increase in the risk of 180-day mortality [47]. Among patients with cirrhosis, gut dysbiosis identified upon admission was associated with progression to organ failure, transfer to the ICU, and death independent of other clinical risk factors [48]. Such low baseline microbiome diversity in the critically ill raises many important questions, such as nutritional status of patients, comorbidities, and whether low baseline gut microbiome diversity may be one of several risk factors contributing to the pathogenesis of the critical illness itself.

While previous studies have reported that fermented foods can enhance gut microbiome diversity [33–37], our study found that a single serving of kefir did not have this effect in our critically ill cohort in the short span of ~ 72 h. This result is not entirely surprising, given that our assessment centered around the changes in α-diversities within a short 72 h window of kefir supplementation. A span of 72 h might be insufficient to observe significant shifts in the microbiome composition [49, 50], especially considering that most of our patients were on antibiotics.

Interestingly, of the 12 microbial species present in the kefir, three species (*Lactobacillus plantarum*, *Lactobacillus reuteri*, and *Lactobacillus rhamnosus*) were more frequently detected in gut microbiome samples at T2 (Fig. 4). This indicates that, within a 72 h window, kefir administration was able to introduce certain beneficial microbes into the gut environment of ICU patients, despite their concurrent medication regimens, including antibiotics. However, *Bifidobacterium longum*, another microbial species in the administered kefir, showed decreased prevalence in patients by T2. While the exact reason for this is unknown, it is conceivable that *Bifidobacterium longum* might be particularly susceptible to certain medications, potentially antibiotics, given to ICU patients.

While patients exhibited a decrease in gut microbial diversity during their ICU stay, the significant increase in their GMWI within 72 h following kefir administration is an exciting finding of our study (Fig. 5a). As illustrated in Fig. 5a, the GMWI score improved at T2, largely due to the reduction of health-scarce (disease-associated) GMWI microbial species from T1. This drop in many of the health-scarce species by T2 might be attributed to antibiotic treatments that eliminated a range of species present in patients’ gut upon admission to the ICU.

Several limitations of our study should be noted when interpreting our results. First, being a feasibility study, there was no control group for comparison of outcomes. Second, more than half of the recruited patients did not have a bowel movement while they remained in the ICU, so a stool sample could not be collected. Only 13 patients out of the 54 recruited provided more than one stool sample during our collection time points. This situation highlights the inherent challenges in conducting research that depends on voluntary, non-invasive biological sample collection from ICU patients, thereby limiting the scope of conclusions that can be derived from our data. Third, we did not implement any specific adjustments for missing data although we were only able to gather a limited number of stool samples at a second timepoint. Considering the pilot nature of the study and the novelty of examining gut microbiome changes following kefir use in the critically ill, it would have been impractical to attempt imputation for any missing follow-up data. Fourth, due to the nature of our work as a pilot study, it was not feasible to accurately estimate the required sample size for our study through a statistical power analysis. This study was pioneering in its approach, and the variance in the patient population could not be predetermined, as there were no existing assumptions to rely on. Nevertheless, we believe that our sample size was sufficient to address this objective. Certainly, the data obtained from this study will be essential in providing a more precise sample size estimation for future randomized trials aimed at determining efficacy, benefiting both our research and that of others in this field. Last, it was not possible to control for the influence of antibiotics in this single-arm pilot study. However, it is noteworthy that all patients from whom we were able to collect two stool samples had received antibiotics with anaerobic coverage. Therefore, given this uniform treatment background across the study cohort, antibiotics were not regarded as a confounding factor for the observed changes in the gut microbiome from the first to the second stool sample.

Despite the limitations mentioned above, our study represents the first of its kind to administer kefir to critically ill patients in an ascending dose regimen, coupled with close monitoring for potential adverse effects and tolerability. Future research should focus on optimizing the dosage of kefir, enhancing sample collection methods, and incorporating a control group. These improvements are crucial for a more comprehensive evaluation of kefir’s safety, tolerability, and its potential in mitigating gut dysbiosis. Such advancements could significantly contribute to improving patient outcomes in the ICU environment.

## Conclusions

Our study confirms that kefir can be safely and feasibly administered to critically ill patients with a functional gastrointestinal system. Our findings highlight the notable lack of gut microbiome diversity in patients upon ICU admission. Given the well-established links between gut dysbiosis and adverse patient outcomes, there is an imperative need to continue focusing on enhancing the gut microbiome of patients in the ICU.

### Supplementary Information

**Additional file 1: Table S1.** Nutrition and ingredient information for a serving (240 mL or 8 oz) of kefir. **Table S2.** List of health-prevalent and health-scarce species used to calculate the Gut Microbiome Wellness Index (GMWI).

## Data Availability

Sequencing data for stool metagenomes used in this study have been deposited at NCBI’s Sequence Read Archive (SRA) data repository (BioProject number PRJNA1016439). The deposited sequences include .fastq files for 42 stool metagenomes collected from 29 critically ill patients admitted to the ICU. Human reads were removed prior to data upload to NCBI SRA.

## Abbreviations

Apache: Acute Physiology and Chronic Health Evaluation
BMI: Body mass index
EHR: Electronic health record
FDA: Food and Drug Administration
GMWI: Gut Microbiome Wellness Index
HIPAA: Health Insurance Portability and Accountability Act
Hospital_LOS: Length of stay in the hospital
ICU: Intensive care unit
MICU_LOS: Length of stay in medical intensive care unit
PERMANOVA: Permutational multivariate analysis of variance
PCoA: Principal coordinate ordination analysis
TSS: Total sum-scaling
