# Supplementary material for: Safety, feasibility, and impact on the gut microbiome of kefir administration in critically ill adults
Source: BMC Med. 2024 Feb 20;22:80. doi: 10.1186/s12916-024-03299-x (PMC10880344; doi:10.1186/s12916-024-03299-x)
Supplement: Supplementary file 1 — Additional file 1: Table S1. Nutrition and ingredient information for a serving (240 mL or 8 oz) of kefir. Table S2. List of health-prevalent and health-scarce species used to calculate the Gut Microbiome Wellness Index (GMWI). [file 12916_2024_3299_MOESM1_ESM.docx]

**Table S1. Nutrition and ingredient information for a serving (240 mL or 8 oz) of kefir.**

|  | Original kefir (unsweetened) | Kefir with vanilla flavor | Kefir with strawberry flavor | Kefir with mixed berry flavor |
| --- | --- | --- | --- | --- |
| Energy (calories) | 150 | 140 | 140 | 140 |
| Total fat (g) | 8 | 2 | 2 | 2 |
| Saturated fat (g) | 5 | 1.5 | 1.5 | 1.5 |
| Cholesterol (mg) | 30 | 10 | 10 | 10 |
| Sodium (mg) | 125 | 125 | 125 | 125 |
| Total carbohydrates (g) | 12 | 18 | 18 | 18 |
| Sugars (g) | 12 | 18 | 18 | 18 |
| Added sugars (g) | 0 | 8 | 8 | 8 |
| Protein (g) | 8 | 10 | 10 | 10 |
| Vitamin A (µg, % of daily need) | 110, 10% | - | - | - |
| Vitamin D (µg, % of daily need) | 5, 25% | 3.8, 20% | 3.8, 20% | 3.8, 20% |
| Calcium (µg, % of daily need) | 397, 30% | 390, 30% | 390, 30% | 390, 30% |
| Potassium (µg, % of daily need) | 376, 8% | 380, 8% | 380, 8% | 380, 8% |
| Ingredients | Pasteurized cultured milk, pectin, vitamin D3 | Pasteurized low fat milk, cane sugar, nonfat milk, natural vanilla flavor, natural flavors, pectin, vitamin A palmitate, vitamin D3 | Pasteurized low fat milk, cane sugar, nonfat milk, natural strawberry flavor, natural flavors, pectin, vegetable juice (for color), vitamin A palmitate, vitamin D3 | Pasteurized low fat milk, cane sugar, nonfat milk, natural strawberry flavor, natural raspberry flavor, natural blueberry flavor, natural flavors, pectin, vegetable juice (for color), vitamin A palmitate, vitamin D3 |
| Live & active probiotic cultures^*^ | Included | Included | Included | Included |

**Bifidobacterium lactis*, *Lactobacillus lactis*, *Saccharomyces florentinus*, *Streptococcus diacetylactis*, *Lactobacillus acidophilus*, *Bifidobacterium longum*, *Lactobacillus casei*, *Lactobacillus reuteri*, *Lactobacillus plantarum*, *Lactobacillus rhamnosus*, *Bifidobacterium breve*, and *Leuconostoc cremoris*.

**Table S2. List of health-prevalent and health-scarce species used to calculate the Gut Microbiome Wellness Index (GMWI).**

| Health-prevalent species | Health-scarce species |
| --- | --- |
| *Alistipes inops* | *Anaerotignum lactatifermentans* |
| *Bacteroides sp.* CAG 144 | *Bifidobacterium dentium* |
| *Bifidobacterium catenulatum* | *Blautia hansenii* |
| *Butyrivibrio crossotus* | *Blautia producta* |
| *Catenibacterium mitsuokai* | *Clostridium bolteae* |
| *Clostridium sp.* CAG 167 | *Clostridium bolteae* CAG 59 |
| *Clostridium sp.* CAG 253 | *Clostridium clostridioforme* |
| *Coprococcus eutactus* | *Clostridium symbiosum* |
| *Firmicutes bacterium* CAG 110 | *Dialister pneumosintes* |
| *Firmicutes bacterium* CAG 170 | *Erysipelatoclostridium ramosum* |
| *Firmicutes bacterium* CAG 95 | *Fusobacterium mortiferum* |
| *Lactobacillus ruminis* | *Fusobacterium nucleatum* |
| *Roseburia sp.* CAG 182 | *Hungatella hathewayi* |
| *Roseburia sp.* CAG 309 | *Klebsiella quasipneumoniae* |
| *Victivallis vadensis* | *Klebsiella variicola* |
|  | *Lactobacillus salivarius* |
|  | *Megasphaera micronuciformis* |
|  | *Parvimonas micra* |
|  | *Peptostreptococcus stomatis* |
|  | *Sellimonas intestinalis* |
|  | *Streptococcus anginosus* group |
|  | *Streptococcus gordonii* |
|  | *Streptococcus infantis* |
|  | *Streptococcus mitis* |
|  | *Streptococcus oralis* |
|  | *Streptococcus vestibularis* |
|  | *Tyzzerella nexilis* |
